# A Cohort Study Risk Factor Analysis for Endemic Disease in Pre-Weaned Dairy Heifer Calves

**DOI:** 10.3390/ani11020378

**Published:** 2021-02-02

**Authors:** Kate F. Johnson, Natalie Chancellor, D. Claire Wathes

**Affiliations:** Department of Pathobiology and Population Sciences, Royal Veterinary College, Hawkshead Lane, North Mymms, Hatfield, Herts AL9 7TA, UK; k.f.johnson@reading.ac.uk (K.F.J.); nchancellor@rvc.ac.uk (N.C.)

**Keywords:** calf, heifer, dairy, diarrhoea, bovine respiratory disease, umbilical disease, colostrum, milk, IGF-1

## Abstract

**Simple Summary:**

Most dairy heifer calves are removed from their dam and reared on milk from birth until weaning at around nine weeks of age. During this period they are susceptible to diseases which reduce their welfare and later performance in the dairy herd and can cause mortality. This study investigated the risk factors for disease on 11 UK dairy farms. Each calf received a weekly clinical examination. Out of 492 heifers recruited, diarrhoea, bovine respiratory disease (BRD) and umbilical disease were recorded in 48.2%, 45.9% and 28.7%, respectively. This was assessed using a composite disease score (CDS), reflecting severity and duration. The CDS for diarrhoea decreased when more calves were born in the same week, but this increased the risk of umbilical disease. The CDS for BRD was reduced by housing calves in fixed groups and feeding them more milk. Being born at a warmer time of year reduced the severity of BRD but increased it for umbilical disease. Calves acquire their initial immunity by ingesting antibodies in colostrum. Better immunity reduced the severity of BRD but failed to protect against diarrhoea or umbilical disease. Calves with a higher circulating concentration of the metabolic hormone insulin-like growth factor 1 (IGF-1) experienced less severe disease. Providing farmers and veterinarians with a better understanding of such risk factors helps them to improve their management practices to reduce disease incidence.

**Abstract:**

Dairy heifer calves experience high levels of contagious disease during their preweaning period, which may result in poor welfare, reduced performance or mortality. We determined risk factors for disease in a cohort study of 492 heifers recruited from 11 commercial UK dairy farms. Every animal received a weekly examination by a veterinarian from birth to nine weeks using the Wisconsin scoring system. Multivariable models were constructed using a hierarchical model with calf nested within farm. Outcome variables for each disease included a binary outcome (yes/no), disease duration and a composite disease score (CDS) including both severity and duration. Diarrhoea, bovine respiratory disease (BRD) and umbilical disease were recorded in 48.2%, 45.9% and 28.7% of calves, respectively. A higher heifer calving intensity in the week of birth reduced the CDS for diarrhoea, with a marginal benefit of improved passive transfer (serum immunoglobulin G (IgG) measured at recruitment). The CDS for BRD was reduced by housing in fixed groups, higher mean temperature in month of birth, increasing milk solids fed, increasing IgG, and higher plasma IGF-1 at recruitment. Conversely, higher calving intensity and higher temperature both increased the CDS for umbilical disease, whereas high IGF-1 was again protective. Although good passive transfer reduced the severity of BRD, it was not significant in models for diarrhoea and umbilical disease, emphasising the need to optimise other aspects of management. Measuring IGF-1 in the first week was a useful additional indicator for disease risk.

## 1. Introduction

In the preweaning period, dairy heifer calves have the highest risk of contagious disease and death, making this period a crucial time to manage on-farm morbidity and mortality. However, studying the risk factors for good health and performance on dairy farms can be challenging for several reasons. Firstly, heifers need to be checked regularly to assess parameters relevant to the perinatal period (e.g., birth weight and passive transfer of immunity) as well as health and growth rates. The typical duration of diarrhoea is only one week with respiratory disease often lasting two to three weeks [1]. Furthermore, relying on farm records reduces the sensitivity of diagnosis. Many farmers are taught to recognise animals refusing milk as a sign of disease, whereas studies demonstrate that calves on a restricted milk ration are unlikely to refuse food even with diarrhoea or respiratory disease [2,3]. Another challenge with understanding the risk factors for dairy heifer performance is the high variability seen within and between farms [4,5,6], meaning that large cohorts are required to find meaningful results. This, combined with the necessity for trained technicians or vets to recognise disease, is required to achieve adequate sensitivity.

Disease incidence is known to be high in dairy heifer calves, with diarrhoea and bovine respiratory disease (BRD) being the most important diseases worldwide [7,8]. Umbilical infections are another common problem [9,10]. Such diseases during the preweaning period lead to decreased feed conversion efficiency [11,12], reduced growth rates [13] and impaired later performance as productive dairy cattle [13,14] as well as being the leading causes of death in heifer calves [8]. In addition to having economically important impacts on calf performance and mortality, they also cause poor animal welfare [15,16] and have a negative impact on farmer’s perceptions and job satisfaction [17]. Disease prevention is consequently a vital part of heifer management.

A wide range of management strategies are employed when rearing heifer calves, with major differences in housing, nutrition and group sizes [18]. These are known to impact the risk of disease at a farm level [6]. In practice, however, there is both intentional and unintentional variation in how farm policies are applied, meaning that it is also informative to investigate how management affects disease risk on an individual calf basis. There may also be intrinsic calf factors involved, such as breed, size and dam factors that impact on the risk of disease. The aim of this study was to assess calf health on a number of farms using weekly health checks performed by a veterinarian from birth to nine weeks old. Management practices including those related to feeding, housing and assessment of passive transfer (blood immunoglobulin (IgG) and total protein (TP) [10]) as applied to the individual calf were also recorded. In addition measurements of the circulating concentration of insulin-like growth factor 1 (IGF-1) were made to assess metabolic status [4]. The time span investigated covered the preweaning period, although some calves were weaned at an earlier time point. Potential risk factors associated with diarrhoea, BRD and umbilical disease were then assessed using multivariable models.

## 2. Materials and Methods

### 2.1. Cohort Design and Recruitment, Herd Profiles and Management

A convenience sample of eleven dairy herds in southeast England with a herd size of between 200 to 550 adult cows was recruited. All farms were visited weekly to recruit all newly born heifer calves alive after 24 h. These visits continued until between 26–56 calves had been recruited per farm, giving a final cohort size of 492. Details of the farm management, colostrum management and disease rates by farm have been published previously [6]. This paper provides an analysis of the risk factors for disease on an individual calf basis. A literature review revealed that whilst little was known of calf disease rates in the UK, the incidences of diarrhoea and BRD were usually between 10–25% and 7–29% respectively in other European and American countries [19]. Assuming a rate for each of these diseases of around 20%, a target cohort of 500 was considered adequate to allow for subsequent assessment of risk factors that changed disease incidence. Power calculations were completed using a population rate of 20% and with the ability to detect a 5% increase or decrease in rates of contagious disease as a binomial endpoint. To detect 15% occurrence with an α of 0.05, a β of 0.1 and a power of 0.9, a sample size of 419 is required; for a 25% occurrence rate a sample of 503 would be required [20]. The study was performed under licence from the UK Animals (Scientific Procedures) Act 1986 with approval by the RVC Ethical Review Process.

### 2.2. Farm and Dam

An initial interview with the herdsmen using a standardised data capture template was used to obtain information on calf rearing practices and this was updated with additional information on individual calves and their dams during the weekly visits. The variables used for the subsequent analyses are detailed in Supplementary Table S1. Dam data included any incidences of perinatal disease or dystocia requiring calving assistance. These were both scored on a binary scale (yes/no). The breed of calf was recorded as being Holstein, Friesian, Jersey, Ayrshire, Swedish Red, or a cross between two of these breeds. Herd calving pattern was classified as all year round (*n* = 8) or autumn block calving (*n* = 3).

### 2.3. Calf Husbandry

Heifer calving intensity was recorded as the number of heifers born on that farm in the week of the calf’s recruitment. Mean temperature in the month the calf was born was accessed from the UK government weather statistics [21]. Protocols for calf housing were recorded, including group sizes, any transfers between pens and the age at which this occurred. At every visit the calves present in each group were noted. If calves changed groups they were classified as “continuous flow” whereas those which remained in a stable group throughout the preweaning period were classified as being in a fixed group. The breed of calf and its age at disbudding were also noted.

### 2.4. Calf Nutrition

Any calf receiving colostrum by bottle or oesophageal tube was recorded. Otherwise they were assumed to have received colostrum directly from the dam. After separation from the dam all farms fed transitional waste milk when available, with all farms allocating each calf 2 L twice daily. Details of the subsequent milk feeding and weaning strategy on each farm were recorded as described previously [22]. In brief, milk feeding was classified as bucket, teat or automated and frequency was recorded as once, twice or thrice daily or continuously available. All farms fed warm milk in the approximate temperature range 35–40 °C. The type of milk was either waste milk from the herd or milk replacer, for which brand details and mixing rate were noted. Age at weaning was recorded on an individual calf basis. All farms used a step down process for weaning with the timing classified as being based on a fixed plan, or based on calf age and/or condition. This information was used to estimate the actual milk consumed by each individual calf in their first five weeks (1–35 days) and subsequently (35–63 days) as described previously [22]. For calves fed from drums, the milk consumption was averaged across the group. Information was also obtained on concentrate feeding (which was always ad libitum) and availability of roughage (hay, silage, straw in feeder or straw as bedding).

### 2.5. Collection of Morphometric Data

Calf height at the withers, trunk length and girth behind the forelimb were recorded at recruitment (week 1, 4.1 ± 2.2 days), in week 5 (37 ± 6.0 days) and week 9 (60 ± 4.0 days). In preweaning heifers, calf girth is an acceptable proxy for weight (r^2^ = 0.89, *p* < 0.001), so weights were calculated as described previously [22] using Formula (1):

Weight (kg) = (Girth (cm) × 1.96) − 113.(1)

Ponderal index in kg/m^3^ was calculated using Formula (2):

Weight (kg)/(height (m) + length (m))^3^ (2)

This determines the relationship between mass and skeletal size, providing a measure of leanness [22].

### 2.6. Measurement of Passive Transfer and Circulating IGF-1

Jugular vein blood samples were collected from each calf at recruitment in week 1 into a plain vacutainer (BD, Oxford, UK), centrifuged after clotting and the serum stored at −18 °C for later analysis. A refractometer (RHC-200, Huake Instrument Co. Ltd., Shen Zhen, China) was calibrated with distilled water then used to assess serum TP. IgG in the same sample was measured using a radial immuno-diffusion assay kit (VMRD, Pullman, WA, USA) according to the manufacturer’s instructions. Heparinised blood samples were collected at weeks 1 and 5, centrifuged (2000× *g*, 10 min, room temperature) to collect plasma and IGF-1 was measured using a commercial ELISA kit (IDS, Tyne and Wear, UK) as described previously [23].

### 2.7. Calf Health Monitoring

Calf health was assessed via clinical examination by the same veterinarian at weekly visits from 1–9 weeks of age as described in detail previously [6]. The scoring used a modified version of that developed at the University of Wisconsin by McGuirk [24]. Bovine respiratory disease (BRD) was assessed using: (i) temperature, with cut-off values of 38.5 °C, 39.0 °C and 39.5 °C; (ii) ocular and nasal discharges and (iii) the presence of induced or spontaneous cough. These were all scored on a scale from 0–3. Heifers with a total combined score from these three measurements ≥5 were classified as having BRD. Faecal consistency was given a score of 0, normal; 1, pasty; 2, loose or 3, watery and calves with a faecal score ≥2 were classified as having diarrhoea. Notes were also taken at each visit to record any other health problems. The most common were umbilical infections which were scored as: 0, no infection; 1, local swelling <5 cm diameter; 2, local swelling >5 cm diameter or 3, local swelling with signs of systemic disease of phlebitis, polyarthritis or peritonitis. For ethical reasons, both the farmers and their local veterinary surgeon were informed of any animals showing clinical signs of disease after each visit.

Treatment protocols for disease were agreed with each farm manager before the start of the study to ensure that sick animals received appropriate, licenced treatments. For BRD this always included a licenced antibiotic and a non-steroidal anti-inflammatory drug. For umbilical disease, all farms used licenced antibiotics and some farms also used a non-steroidal anti-inflammatory drug, depending on the protocols agreed with their veterinary surgeons. The farm medicines books (a legal requirement) were checked weekly to determine which calves had been treated for BRD or omphalophlebitis. For diarrhoea, the treatment protocol used rehydration solutions. As these do not legally have to be recorded, it was not possible to obtain exact information about which calves were fed rehydration solution or how many times they were treated. Where farmers described having treated calves but there were no signs of any disease on clinical examination, the clinical signs recorded by the farmer were used. All calf deaths were recorded and the presumed cause was established by the local veterinarian by means of a gross pathology post mortem examination.

### 2.8. Data Analysis and Univariate Statistics

Data were stored in Excel and arranged into a relational database using Access (Microsoft Office, Microsoft, Redmond, WA, USA). All analysis was completed in R using the lattice package for graphics (http://www.r-project.org). Disease was measured in each calf as described above using a systematic scoring system each week for nine weeks. Incidence rates for diseases were calculated as the number of new cases divided by calf weeks at risk. New cases were defined as a calf meeting the diagnostic criteria that had been scored as healthy for that condition in the previous week. For incidence rate calculations, this meant that repeated cases were included but continuing disease was not. The calculations of how many weeks a calf had a disease used the total number of positive clinical examinations and did not account for difference between long disease duration compared with repeated cases. The three diseases selected for analysis were BRD, diarrhoea and umbilical disease. Three different outcome variables for each of these diseases over the course of the nine weeks were assessed: (1) the presence or absence of disease over the entire period (used as an ordinal variable); (2) the number of weeks when the calf was found with disease, or (3) the total score from each week summed to give a composite disease score (CDS). This last variable increased with both increasing disease duration and increasing severity of clinical signs, potentially providing additional information about calf health. In order to compile the CDS for BRD, the calf must have had a score of at least 5 (the threshold for diagnosis) in a particular week. In any week where a calf was below this threshold, it was allocated a score of 0, whereas in any week above the threshold the score minus 4 was used (giving a value of at least 1). This system was devised to avoid the possibility of calves with variable healthy scores below 5 being confused with animals which experienced at least one week above the threshold for BRD diagnosis.

All the candidate variables considered are summarised in Supplementary Table S1. Each variable was initially tested for normality and transformed if necessary. Results are presented as either mean (±SD) or count data and the percentage. Variables were then plotted against the outcome in turn and tested in univariate analysis. Post hoc testing was completed using the Tukey HSD method in the R package “agricolae” to correct for multiple testing. Linear regression was used to assess the relationships between continuous variables using the “l m” function in R. To calculate marginal means for the probability of an event based on model outcomes, the variable of interest was imputed from its first to 99th percentile. All other continuous covariates were held at their median value and any categorical covariates at their referent value.

### 2.9. Multivariable Statistics

For binary outcomes, generalised linear models were used with formula (3), including farm as a random effect:

Binary outcome = constant + β[variable of interest] + Z[farm].(3)

For continuous outcomes, linear models were fitted with formula (4) including farm as a random effect:

Continuous outcome = constant + β[variable of interest] + Z[farm].(4)

The results were recorded with the effect estimate and standard deviation, the z (binary) or t (continuous) values for each intercept and variable and the *p* values. All multivariable models included farm as a random factor, using a hierarchical model with calf nested within farm. An identity covariance structure was used, as farms were assumed to be independent from each other. Models were fitted using the lmer function in the package lme4 with the lmerTest package used to calculate *p*-values for each variable (https://cran.r-project.org/web/packages/lmerTest/index.html). Variables were initially considered for inclusion in the multivariate linear mixed effects models if *p* < 0.2 in univariate analysis. To remove covariance between fixed effects recording related information (e.g., serum TP and serum IgG, r^2^ = 0.71), only the variable with the highest model r^2^ was included. All remaining candidate variables were tested in a multivariate model with a backwards stepwise approach using the “anova(model1, model2)” function in R. This uses likelihood ratio tests to assess differences between two nested models and displays the Akaike information criterion (AIC) and the Bayesian information criterion (BIC), providing measures of relative model quality. The significantly best model was taken forward. Where there was no significant difference, the model with the lower AIC and BIC was selected. All variables that did not improve the model were excluded to give a final model. The effect estimate, the 95% confidence intervals (using the Wald procedure) and the *p* value were calculated.

## 3. Results

The descriptive statistics for each variable tested and any transformations used on the data are detailed in Supplementary Table S1. For all three diseases considered, the results of the generalised linear mixed effects model used for the outcome variables weeks with disease or CDS gave very similar outputs, so only the CDS models have been reported. The rates of disease incidence recorded exceeded the sample size required by our power calculations and meant that the statistical tests had adequate power to detect differences in disease incidence.

### 3.1. Correlated Variables

**Figure 1:**
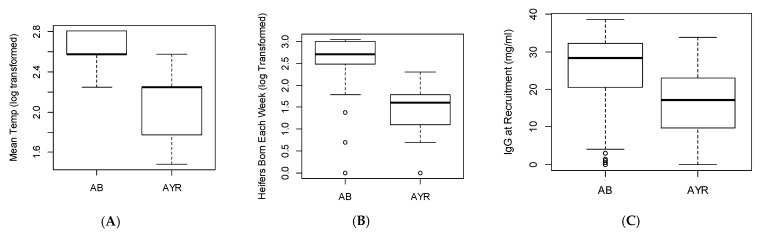
Box and whisker plots showing significant relationships between calving pattern (autumn block, AB, *n* = 3 farms or all year round, AYR, *n* = 8 farms) and other variables tested. (**A**) Mean temperature in the month of calf birth. (**B**) Calving intensity (number of heifers born in the same week, analysed and plotted on a logarithmic scale) and (**C**) passive transfer measured by serum immunoglobulin (IgG) at recruitment. These were all highly significant in a linear model (*p* < 0.001). Boxes show the median, 25th and 75th centile, whiskers indicate the minimum and maximum values, with ○ as outliers.

Calving pattern was highly correlated with the heifer calving intensity and the mean temperature in the month when a heifer was born, which were both significantly higher in the farms with an autumn block (AB) calving pattern (*n* = 3) compared to the AYR calving farms (*n* = 8) (Figure 1). The small sample size for “farm” limits how reliably these data can be interpreted, but it was necessary to include these variables in the models because they had large impacts on the outcomes data. Consequently, these data are reported here to aid interpretation of the main results.

No AYR farm had more than eight calves born in the same week whereas >90% of AB calves were born in groups of >10. Only one of these variables could therefore be included in any model without convergence errors. In each case where all three of these variables were associated with the outcome, only the variable which most improved the model was included in the final model. The correlation between passive transfer (measured as serum IgG) and calving pattern was also highly statistically significant. The r^2^ was 0.17, meaning that 17% of the variation in IgG at recruitment could be explained by the calving pattern. This level was not high enough to cause convergence errors in the multivariate models. However, in several models where both IgG and calving pattern were correlated with an outcome of interest in univariate analysis, then only one would have a significant association in the multivariate model, suggesting confounding. In these cases, as described in the methods, variables were excluded if they did not improve the model.

### 3.2. Diarrhoea

During the first nine weeks of life 255/492 (48.2%) of calves were diagnosed with diarrhoea. Most cases were seen in the first month, peaking in week 2 when 126 calves (28.2%) reached the threshold for diagnosis. Subsequently fewer than 3% of calves over four weeks old were observed with diarrhoea in any week. The overall incidence rate was 7.8 cases/100 calf weeks at risk (95% CI: 7.1–8.8).

Calves with only one week of diarrhoea had a CDS of either 1 (142/180, 78.8%) or 2 (38/180, 21.2%). Calves with two weeks of disease had a CDS between 2 and 4, and those with three weeks of disease had a CDS of between 3 and 5. Very few calves (*n* = 4 in total) were affected with diarrhoea for more than three weeks. Results of a generalised linear mixed effects model for having diarrhoea in weeks 1 to 9 (yes/no) showed that the risk was associated with calving pattern and calf breed (Table 1). Jersey calves were only one fifth as likely to have diarrhoea compared to Holstein calves, whilst in calves born in herds with an AYR calving pattern the odds were increased by 73% compared with calves born in AB calving herds. These results should, however, be interpreted with caution as the intercept in this model had a nonsignificant *p* value and it returned several convergence warnings.

The linear mixed effects model for the composite diarrhoea score demonstrated that heifer calving intensity was significantly associated with disease and there was also a trend for increasing IgG at recruitment to reduce disease risk (Table 2).

**Figure 2:**
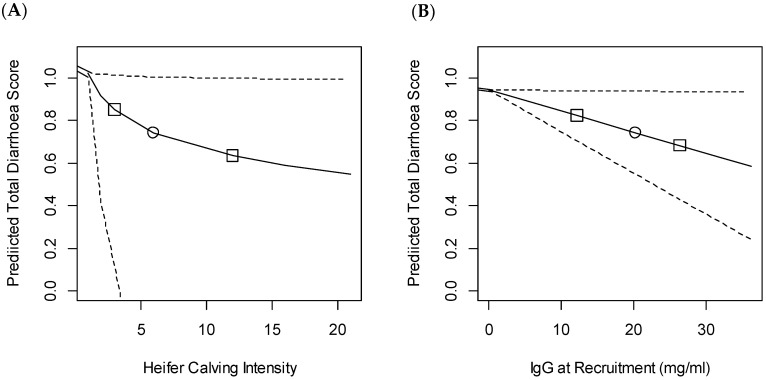
Predicted composite diarrhoea score above the threshold for diagnosis in the first nine weeks based on variation in (**A**) heifer calving intensity (number of heifers born in the same week on the same farm as each individual) and (**B**) serum immunoglobulin (IgG) at recruitment (mg/mL). The study included 492 heifers recruited from 11 farms. The predicted score was calculated using the generalised mixed model output described in Table 2. For each figure median values of the other variables was assumed, which were: IgG at recruitment, 20.2 mg/mL; calving intensity, six heifers born in the same week. Effect estimates were then calculated based on varying only the variable of interest from the first to the 99th percentile. Dashed lines show 95% confidence intervals, ○ indicates the median value and □ the 25th and 75th centiles.

Both of these variables had a large impact on the expected disease score (Figure 2). Heifer calving intensity was log transformed in the model, leading to a curved relationship and asymmetric confidence intervals. An increase from the fifth to 95th percentiles (1–20 calves born in the same farm per week) was associated with a decrease of 0.47 in the composite diarrhoea score. An increase in IgG at recruitment from the fifth to 95th percentiles (0–33.8 mg/mL) was associated with a decrease in the CDS of 0.34. However, the 95% confidence intervals for the effect of serum IgG overlapped with zero, suggesting that there was between a 5% to 10% possibility that the change observed could be due to chance.

### 3.3. Bovine Respiratory Disease

A similar proportion of calves (266/492, 45.9%) experienced BRD during their first nine weeks. The pattern was, however, different to that for diarrhoea, with cases spread throughout this period. The overall incidence rate was 10.1 cases/100 calf weeks at risk (95% CI: 9.2–11.2). For BRD there was also a wider range of CDS observed. Animals with only a single week of disease had scores between 1 and 5 and calves with two or three weeks of disease had total scores between 2 and 10. Fifteen calves had BRD for between four and six weeks and these had a CDS of between 4 and 15. The previous occurrence of diarrhoea was tested as a risk factor in the models for BRD, but no association was found.

**Figure 3:**
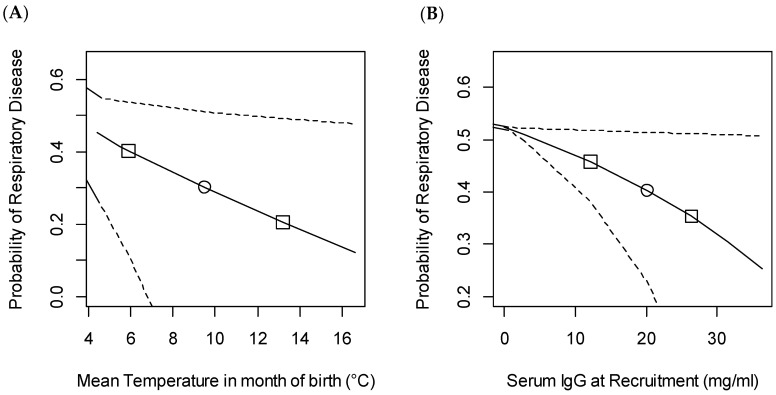
Predicted probability of bovine respiratory disease (BRD) preweaning based on variation in (**A**) mean temperature in the month of birth and (**B**) IgG at recruitment (mg/mL). The marginal mean probability of respiratory disease was calculated for the observed range of the variables with all other covariates held constant at their median value. The study included 492 heifers recruited from 11 farms. Dashed lines show 95% confidence intervals, ○ median value, □ 25th and 75th centile.

The generalised linear mixed model found several variables associated with the risk of contracting BRD in the first nine weeks of life (Table 3, Figure 3).

There was a trend for breeds other than Holstein to have less respiratory disease than Holstein calves. Housing calves in fixed groups significantly reduced the odds of BRD, with calves in stable groups being 0.4 times less likely to contract disease compared with those in continuous flow systems. This would give a probability of disease preweaning of 0.34 compared to 0.46 in the population as a whole. Warmer weather in the month of birth was associated with decreased likelihood that calves would catch BRD. As the temperature increased from 4.4 to 16.6 °C (fifth to 95th percentiles) this was associated with a decrease in the probability of disease reducing from 0.45 to 0.12. Improved passive transfer was also protective, with an increase in serum IgG at recruitment from 0 to 33.8 mg/mL (fifth to 95th percentiles) associated with a decrease in probability of disease from 0.52 to 0.28.

**Figure 4:**
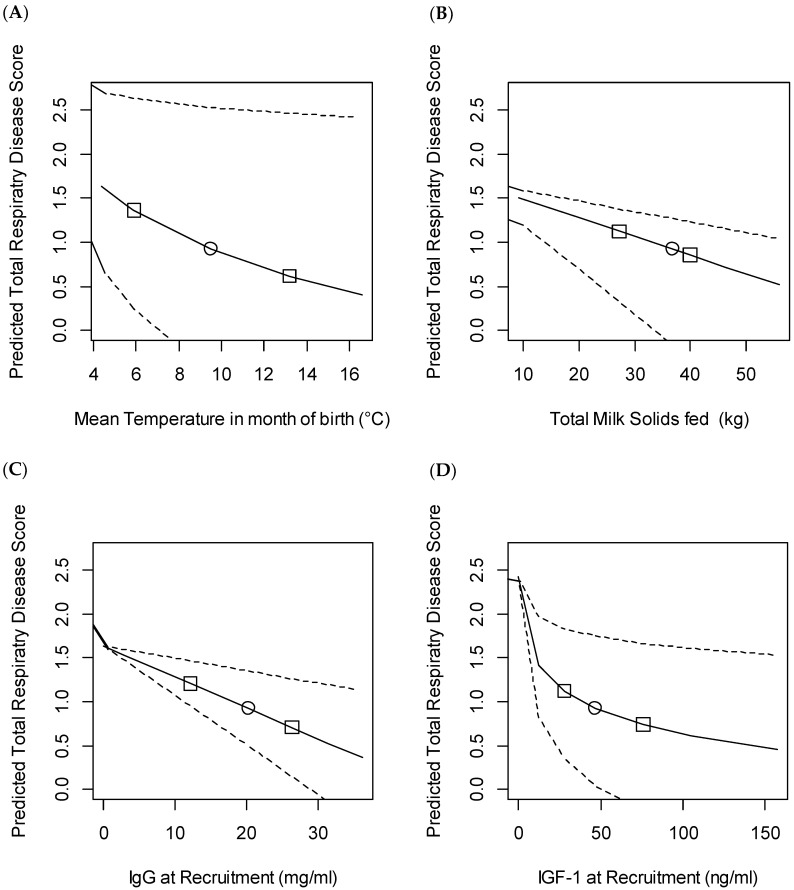
Predicted composite bovine respiratory disease (BRD) score above the threshold for diagnosis in the first nine weeks of life based on variation in: (**A**) mean temperature in the month of birth; (**B**) total milk solids fed preweaning (kg); (**C**) IgG at recruitment (mg/mL) and (**D**) IGF-1 at recruitment (ng/mL). The study included 492 heifers recruited from 11 farms. The predicted score was calculated using the generalised mixed model output described in Table 4. For each figure median values of all the other variables were assumed and were: mean temperature, 9.5 °C; total milk solids fed, 36.8 kg; IgG at recruitment, 20.2 mg/mL; IGF-1 at recruitment, 47.1 ng/mL. Effect estimates were then calculated based on varying only the variable of interest from the first to the 99th percentile. Dashed lines show 95% confidence intervals, ○ median value, □ 25th and 75th centile.

The linear mixed effects models fitted for the composite BRD score showed that housing calves in fixed groups (compared to continuous flow systems), increasing mean temperature in the month of birth, increasing milk solids fed, increasing passive transfer of immunity from the dam, and increased IGF-1 at recruitment were all associated with a reduced CDS (Table 4). These variables were associated with large changes on the outcome as shown in Figure 4. For example, increasing temperature in the month of birth from 4.4–16.6 °C (5th to 95th percentiles) was associated with a 1.24 decrease in CDS. Similarly, increases in IgG and IGF-1 from the fifth to 95th percentiles were associated with 1.18 and 1.15 reductions in the BRD CDS, respectively. The total milk solids fed was also associated with a reduction in score, although in this case the slope was shallower with a reduction in score of 0.79 predicted when the milk solids fed increased from 16.8–54.1 kg (fifth to 95th percentiles).

### 3.4. Umbilical Infection

Umbilical infection was less common than BRD or diarrhoea. Overall 141/492 (28.7%) of calves were found to have an umbilical infection at some point, with infections diagnosed in calves aged between 1 to 66 days. The median age at diagnosis was 18 days with an interquartile range of 9–27 days. The incidence rate was 6.9 cases per 100 calf weeks at risk (95% CI: 6.6–7.2). In 54 calves (35.5% of those infected), the infection was present for more than one week. Most cases were mild disease, with scores of 1, 2 and 3 awarded on 196, 50 and 3 occasions, respectively. Among calves with disease, 76 received a CDS of 1, 30 a score of 2, and 35 calves had a score of between 3 and 11.

No generalised linear mixed effects model could be fitted for the risk of umbilical infection preweaning because the model failed to converge. The linear mixed effects model for composite umbilical infection score showed significant effects of heifer calving intensity, mean temperature in month of birth and IGF-1 concentration at recruitment (Table 5).

**Figure 5:**
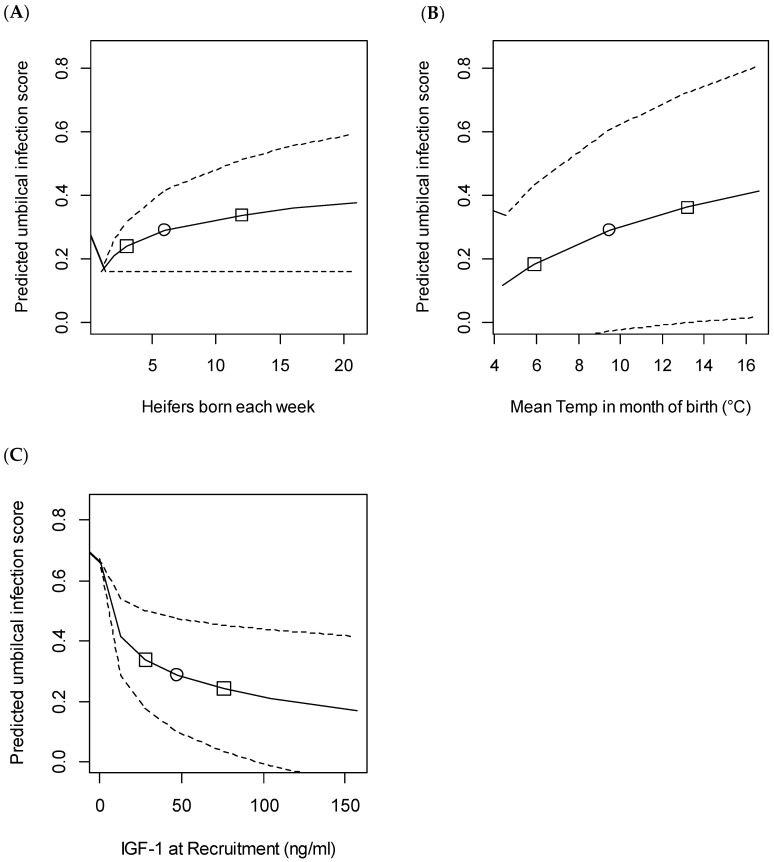
Predicted composite umbilical infection score in the first nine weeks based on variation in (**A**) heifer calving intensity (number of heifers born in the same week on the same farm as each individual); (**B**) mean temperature in the month of birth and (**C**) IGF-1 at recruitment. The predicted score was calculated using the linear mixed model output described in Table 5. The study included 492 heifers recruited from 11 farms. For each figure, median values of all other variables were assumed, which were: calving intensity, six heifers born in the same week; mean temperature, 9.5 °C; IGF-1 at recruitment, 47.1 ng/mL. Effect estimates were then calculated based on varying only the variable of interest from the first to the 99th percentile. Dashed lines show 95% confidence intervals, o median value, □ 25th and 75th centile.

All the associated variables were transformed for analysis, which meant that the figures to illustrate this model all had curved line relationships (Figure 5). Increasing heifer calving intensity from 1 to 20 born per week (5th to 95th percentiles) was associated with a 0.21 increase in the CDS. Similarly, increasing mean temperature in month of birth from 4.4 to 16.6 °C (fifth to 95th percentile) was associated with an increase in score of 0.29. Finally, higher IGF-1 at recruitment was protective and an increase from 5.6 to 124 ng/mL (fifth to 95th percentile) was associated with a fall in CDS of 0.28.

### 3.5. Calf Mortality

The overall mortality rate in the first nine weeks was 22/492 (4.5%), as reported previously (Johnson et al., 2017). The main causes were diarrhoea (*n* = 8) or BRD (*n* = 7). Other causes were dystocia-related (*n* = 2), omphalophlebitis (*n* = 1), neonatal pancytopaenia associated with BVD vaccination of the dam (*n* = 1), rumen bloat (*n* = 2) and accident (*n* = 1). The case fatality rates were 8/308 (2.6%) for diarrhoea, 7/396 (1.8%) for BRD and 1/141 (0.7%) for umbilical disease, respectively.

## 4. Discussion

This study has investigated the risk factors for endemic disease in preweaned heifer calves in the UK. The results confirmed previous work in finding that diarrhoea and BRD were the most prevalent causes of both morbidity and mortality, followed by umbilical disease [25,26]. Disease was recorded by examination from a veterinary surgeon at weekly intervals, known to be a method with good sensitivity [1,27,28]. The use of the Wisconsin scoring system [24] for diagnosis provided reliable information on the incidence and severity of each disease. The use of composite disease scores in the analyses, which accounted for both duration and severity, provided additional information to the use of the individual measures. Using either the number of weeks with disease or the CDS as the outcome variable gave almost identical results for all three diseases considered. In contrast, the binary variable of whether a calf had disease or not did not give consistent results and for both diarrhoea and umbilical infections that model had convergence problems. In the case of BRD it was assumed that more severe symptoms of ocular and nasal discharge, cough and pyrexia indicated more severe respiratory disease. It may be, however, that only one of these symptoms indicates a worse prognosis or a more important long-term impact, rather than the score in general. Another caveat is that the score will be influenced by the day of the visit, which will not necessarily concur with the time of the worst clinical signs. We were also unable to investigate the potential causative pathogens involved, some of which will have differential risk factors.

### 4.1. Passive Transfer

The measurement of IgG by radial diffusion in the first week of life is considered the gold standard to assess passive transfer of immunity from dam to calf. In accord with other studies, the measurement of TP was highly correlated with the IgG measurement [6,29,30] but, in order to avoid covariance, only the IgG concentrations were retained in the models, as these had the highest model r^2^. Failure of passive transfer (FPT) has previously been defined as having circulating levels of IgG less than 10 mg/mL [30]. The incidence of FPT was reported as 26% in a previous survey in the UK [31] and 19% in the USA [32]. In a more recent American study, the proportion of calves with FPT was 12%, with 35.5% of calves having IgG concentrations >25.5 mg/mL [10]. The values reported in our cohort were therefore similar, with 20.7% of calves having an IgG concentration <10 mg/mL and 38.5% having >30 mg/mL. IgG was a significant factor in both the risk of contracting BRD and of reaching a high CDS for this disease. In the case of diarrhoea, there was a trend (*p* = 0.064) towards a protective effect of a high IgG concentration, but IgG was not significant in any of the models relating to umbilical disease. The guidelines relating to beneficial effects of good passive transfer were recently reassessed by a panel of experts [26]. Their consensus agreement was in accord with our findings in that the use of a scale of four serum IgG categories (excellent, good, fair, and poor) at the individual calf level was more informative than setting a single cut-off point to designate failure of passive transfer. The suggested new categories were applied to a longitudinal study of 2360 calves from 104 dairy operations located in the USA [8]. The results showed progressive increases in morbidity (from 28% to 46%) and mortality (from 2.5% to 7.4%) between the excellent and poor passive transfer categories.

The effect of IgG on disease risk in our cohort was, however, smaller than that of the mean temperature in the month of calving or the circulating IGF-1 concentration. The proportion of heifers with BRD preweaning decreased progressively from 55.7% in those with IgG ˂ 10 mg/mL to 44.8% with IgG between 10 and 30 mg/mL and 38.5% of those with IgG ˃ 30 mg/mL. Although this represented a 17% reduction in incidence in animals with the best passive transfer, 38.5% of the calves with an IgG concentration over 30 mg/mL still contracted BRD. The calves in this study had a higher overall prevalence of BRD (45.9%) than in most previous investigations, in which incidence rates of 1–39% have been reported (reviewed by [19]). It is therefore possible that these heifers were in a high pathogen environment which exhausted the limited resource of dam acquired IgG to inhibit respiratory disease, despite good passive transfer. This is supported by another recent UK study in which 37% of dairy heifers contracted BRD even though only 5% had failure of passive transfer [33]. Diagnosis of BRD can be challenging, with farmers known to have poor sensitivity compared to veterinary surgeons and trained technicians [1,34]. Weekly visits and proactive scoring to ascertain disease status are a common experimental technique in both North American cohorts [35,36,37] and European studies [13,33]. Nutrition is also influential, as calves on a low milk ration will be less able to mount an adequate immune response (discussed below). A third possibility could be that some animals were infected with bovine viral diarrhoea virus (BVDV). This was not assessed, but BVDV causes immune suppression and this is known to increase the risk of pneumonia [38,39].

The poor association between passive transfer and diarrhoea incidence was in accord with previous work. A number of earlier cohort studies have demonstrated that passive transfer was not a risk factor for calf diarrhoea [34,36,40,41]. On the other hand, another study found that serum total protein (STP) was associated with diarrhoea in a large study of 2847 animals, although with very poor predictive values [35]. Similarly, diarrhoea incidence was lowest (around 35%) in calves with an STP concentration > 8.5 g/dL in comparison with rates of around 45% in calves with STP < 6 g/dL [25]. Together these results suggest that the protection provided by good passive transfer of immunoglobulin on diarrhoea incidence is relatively small. This is likely to be because the organisms causing scours act locally within the gut rather than systemically and so circulating IgG is not protective, although other constituents of colostrum, such as IgA, or antimicrobial peptides and oligosaccharides may be beneficial [30].

Conversely, effective passive transfer has been clearly associated with reduced umbilical infection in some previous cohorts of dairy heifers [37,42]. Our lack of association was perhaps surprising, although Cortese et al. [25] also failed to show a relationship between STP and the incidences of either septicaemia or joint infection, which may be sequelae of an initial umbilical infection. Some farms in our study used frozen colostrum which might have an adequate IgG content but maternal leukocytes and other factors, such as growth factors would be reduced [30,43]. An alternative potential explanation for this result is that there was insufficient power in our study for it to detect the effect of IgG. There were, however, 141 calves with umbilical infection so the group sizes for both healthy and diseased calves should have been adequate. It is also possible that differences between the physical environment and/or the circulating pathogens in the UK mean that this lack of association represents a real finding. For example, many of the previous studies have taken place in areas with very cold winters (e.g., Scandinavia and Canada), which would reduce the relative humidity and pathogen survival [44].

### 4.2. Maternal Factors and IGF-1

In this study neither perinatal disease in the dam nor dystocia were associated with subsequent disease risk in the calf. This was perhaps surprising as during an assisted or prolonged delivery the calf can be starved of oxygen and become hypoxic and acidotic [45]. This results in reduced vigour and suck reflex, with severely acidotic calves having a much higher risk of postnatal mortality. Assessment for both of these dam factors relied on farm records rather than a direct diagnosis by the attending veterinarian, so it is possible that the data were not completely reliable.

One novel finding of this study was that IGF-1 measured at recruitment was a significant explanatory variable in two of the CDS models. For BRD the variation in IGF-1 had a similar size effect to that attributable to IgG and for umbilical infections only IGF-1 (and not IgG) was significant. IGF-1 is a metabolic hormone which is produced locally in many tissues, with the liver being the main source of IGF-1 in the circulation [46]. It is a potent growth factor and the IGF-1 concentration in juvenile heifers is highly correlated with their growth rate [4,22]. The median concentrations in this cohort of 47 and 52 ng/mL measured at 1 and 5 weeks, respectively, were similar to the average value of 42 ng/mL which we previously reported in dairy heifer calves at one month of age [4]. The IGF-1 concentration present in the circulation in the first week of life is likely to be influenced by both pre- and postnatal effects. Foetal IGF-1 concentrations in late gestation were significantly lower in underfed ewes and were associated with lower circulating levels of foetal plasma glucose and insulin [47,48]. Glucose represents the main supplier of the daily carbon and energy requirements, whilst insulin is a key regulator of tissue accretion and cell proliferation in both bone and soft tissue [49,50]. Postnatally, the IGF-1 concentration was shown to be influenced by nutrient intake in Holstein bull calves fed differing levels of milk replacer [51]. In human babies correlations have been found between low neonatal serum concentrations of IGF-1 with poor brain development and multi-organ morbidities affecting the heart, lungs and gut [52]. In both mouse models and premature human infants reduced IGF-1 concentrations were associated with disrupted lung development, which in turn was thought to have an enduring effect on host resistance to respiratory infections [53]. Having a lower IGF-1 level at 1 month of age also increased the risk of dairy heifer calves dying before six months of age [23]. All of this provides substantial evidence that having low IGF-1 does indicate that a newborn calf is at increased risk of infectious disease and more studies are warranted to understand the main causes and consequences of reduced perinatal IGF-1 concentrations.

### 4.3. Milk Feeding

This study found a highly significant effect of the total milk solids fed preweaning on the composite BRD score. This cohort of animals was well suited to investigating this effect as there was a high incidence of BRD, providing a large sample size, and there was also a large variation in the amount of milk supplied to individual calves due to both different feeding strategies between farms and highly variable weaning ages. Although half of all calves were weaned between 60–69 days of age, the overall range from 34–96 days was much wider and the estimated range of milk solids fed from birth to day 63 was between 16–56 kg [22]. The cohort was recruited from commercial farms rather than being an experimental study on a university farm. This likely contributed to the high variation in the treatment of individuals, such that calves on the same farm often received different actual amounts of milk fed, particularly in their second month. The moderate size of farms in this study with 200 to 550 adult cows might also be more likely to experience variable weaning ages than either very large farms (where batches may be closely grouped by age) or smaller farms, where there may be closer attention paid to individual calves.

There are several possible mechanisms of action for the relationship between more milk feeding and a reduction in respiratory disease. An increased milk ration is associated with reduced calf mortality [3,54]. A review by Khan et al. [12] concluded that it was unlikely that there were specific immune modulatory effects of increasing milk ration and that effects were dependent on overall nutrient delivery. There is indeed good evidence to link impaired immune function directly to a reduction in nutrient availability. Mounting an effective immune response is energetically demanding, as immune cells require an adequate supply of glucose, amino acids, fatty acids and cholesterol/oxysterols in order to function [55,56,57]. Therefore calves on a higher plane of nutrition are likely to have a better response to pathogen challenge, making them less susceptible to prolonged respiratory disease. This is supported by some studies which have shown immune modulatory effects of low milk feeding [58]; for example there was reduced innate immune function in calves fed at low rates [59]. In our study the amount of milk fed during the second month of life had a stronger correlation with disease than milk feeding in either the first month or overall. This could be because most BRD occurred in the second month and it is the milk availability whilst the calf is already unwell which is important. Sickness behaviours include reduced appetite [60,61]. The high palatability of milk may mean that calves with milk available consume more energy. Indeed, calves are known to continue eating all of a restricted milk ration even when unwell [2,3]. This second explanation is supported by the finding that the milk ration was not significant in the risk of contracting BRD in the first place but did influence both the duration and CDS. Having an adequate nutrient supply may therefore help calves to recover more quickly, lessening the impact of disease on their welfare and performance.

### 4.4. Breed

Although the majority of calves in this study (71%) were Holstein, a variety of other pure breeds and cross breeds were present in our cohort, with six out of eleven farms having breeds other than Holstein present. Breed was retained as a factor in two models. Firstly, in the analysis of risk of diarrhoea, purebred Jersey heifers had a lower incidence than all the other breeds, with only 21% as many cases as occurred in the purebred Holsteins. This result should, however, be interpreted with caution as the number of Jersey calves was quite low (*n* = 24) and the intercept in this model had a nonsignificant *p* value and returned several convergence warnings. In the model of BRD incidence the comparison of Holstein versus all other breeds improved the fit of the model and tended towards significance at *p* = 0.097. While it is possible that Holstein heifers do indeed have a higher disease risk, relatively few farms kept different breeds under the same management, making the direct comparisons less reliable. One other recent study was able to compare breed effects more reliably. This was based on a single calf-raising facility which received calves to rear from 15 dairies within two days of their birth, so subsequent management was standardised [25]. In contrast to our data they found that purebred Jerseys (*n* = 2849) had higher incidences of death, diarrhoea, pneumonia and septicaemia in comparison with Holsteins (*n* = 34,135).

### 4.5. Management and Environmental Factors

Management factors which were significant in the various models included calving pattern, calving intensity, and mixing of groups of calves. The average temperature in the month of birth was also influential. Perhaps surprisingly, a higher calving intensity was associated with reduced composite score for diarrhoea as calf scours are known to be associated with cleanliness [62,63]. In this cohort most calves were housed in groups and even those in individual pens were in nose-to-nose contact with other heifers. Higher calving intensity would, however, reduce the range of ages of calves in a single pen, which might reduce disease transmission. This effect might be especially important for the protozoan parasite, *Cryptosporidium parvum,* which takes at least four days to produce infectious oocysts [64]. The opposite effect was seen for umbilical infection in which a higher calving intensity increased the disease score. This might be due to infection pressure, less management time spent on navel disinfection in individual calves or perhaps more navel sucking in larger groups of similarly aged calves.

Calving pattern and temperature in month of calving were confounded, as the temperatures at calving were higher in AB than in AYR calving herds, in which calving continued throughout the winter months. The overall mean monthly temperatures ranged from 4.4 °C to 16.6 °C with a median of 9.5 °C. Higher temperatures reduced both the risk and duration of BRD. Similarly, in a USA-based study, the predicted morbidity risk for calves decreased as the average temperature humidity index increased during the preweaning period [8]. The lower critical temperature in dairy heifers up to three weeks of age is around 15 °C [65], so many calves within the UK are kept at temperatures below their thermoneutral zone. This will directly increase their requirements to use energy from body stores or milk rations to keep warm [66]. In addition, there would also be reduced humidity at higher temperatures, which would affect survival of some airborne pathogen. As with calving intensity, the reverse effect was seen on umbilical infection, with increasing disease scores at higher temperatures. Likely explanations for this include an increased number of flies and environmental pathogen load in the deep straw bedding.

With respect to BRD, the management of the calf housing influenced both the likelihood of catching BRD and its severity score. The risk was reduced by around 40% in heifers kept either individually or in fixed groups compared with those which moved between groups. This is unsurprising as it provides animals which are already infected greater opportunities for transmission through both direct contact and sharing of feeding facilities, particularly when younger animals are placed in with older ones. This supports previous work concluding that successful group housing to avoid BRD requires that calves should be kept in equal age groups which remain stable once they are formed [15]. Curtis et al. [33] reported an increased risk of pneumonia in groups of calves sharing a teat despite receiving more milk, which they attributed to greater pathogen transfer, such as *Mycoplasma* species, between calves. Hepola [67] also reported an association between use of computerised feeding systems and increased incidence of pneumonia.

Previous studies have consistently found that cases of diarrhoea in early life are a risk factor for later BRD [14,68,69]. This relationship was not, however, observed in this cohort. Although it is possible that this was a type one error, the large proportion of calves having contagious disease in our study should have provided sufficient sample sizes to demonstrate this association if it existed. This cohort had very high rates of disease despite fairly good levels of passive transfer. This suggests that the calves were exposed to a high pathogen challenge for both diseases, which might have affected this relationship.

### 4.6. Relevance to Farming Practice

There were very high rates of contagious disease in this cohort with diarrhoea, BRD and umbilical disease recorded in 48.2%, 45.9% and 28.7% of the preweaned calves, respectively. These diseases were previously shown to limit the growth rates for both weight and height [22]. Calf disease also has negative impacts on calf welfare. Pyrexia was used as a diagnostic criteria for respiratory disease and animals with fever exhibit sickness behaviours including reduced feed intake, depressed demeanour, reduced exploratory and play behaviours [2,60,61]. Diarrhoea was the single most common cause of death in this study. This could potentially be reduced by better training of farmers to recognise and treat the symptoms promptly and appropriately. Respiratory disease is recognised as a large welfare and economic burden on dairy heifer rearing [35,70,71]. Severe BRD is also known to affect the long-term performance of that animal in the adult milking herd [5,12,72]. In this study, management factors were found to vary significantly on an individual calf level: even policies which the farmer had control over such as age at disbudding or weaning were highly variable. The latter had a major impact on the amount of milk fed which in turn influenced the severity of BRD. Farmers should therefore be encouraged to feed more physiologically normal amounts of milk and to ensure that every individual calf receives an adequate milk supply.

## 5. Conclusions

Although cleanliness is known to be very important in diarrhoea prevention we found reduced disease with a higher calving intensity when heifer groups were closer in age. There was only a minor beneficial effect of passive transfer. Respiratory disease was reduced by maintaining calves in fixed groups, warmer temperatures, more milk feeding and better passive transfer. The severity of umbilical disease increased with higher calving intensity and temperature. Even calves with good passive transfer were, however, affected by disease. It is important for farmers to realise that passive transfer cannot supply an unlimited number of antibodies and that the protection can be quickly used up in dirty, unhygienic environments in which the calf is exposed to a high pathogen challenge. For both respiratory disease and umbilical infections, IGF-1 in the first week of life may be a good additional indicator to use for disease risk.

## Figures and Tables

**Table 1:** Results of the generalised linear mixed model for variables associated with the risk of dairy heifers (*n* = 492 on 11 farms) showing clinical signs of diarrhoea in their first nine weeks of life. Estimate is exponentiated to give odds ratios for diarrhoea being present.

Variable	Estimate	95% CI	*p*-Value
(Intercept)	0.68	0.42 to 1.1	0.143
Breed—Holstein	(ref)		
Breed—Ayrshire	0.45	0.11 to1.91	0.317
Breed—Friesian	0.60	0.26 to 1.38	0.317
Breed—Friesian × Jersey	0.93	0.26 to 3.29	0.913
Breed—Friesian × Other	0.50	0.05 to 5.14	0.558
Breed—Jersey	0.21	0.06 to 0.81	0.026
Breed—Jersey × Other	0.85	0.22 to 3.32	0.819
Breed—Scandinavian Red×	1.63	0.82 to 3.26	0.215
Calving Pattern—Autumn Block	(ref)		
Calving Pattern—All Year Round	1.73	1.01 to 2.96	0.049

**Table 2:** Results of the linear mixed effects model for the composite diarrhoea score over the threshold for diagnosis in dairy heifers (*n* = 492 on 11 farms) in their first nine weeks of life.

Variable	Estimate	95% CI	*p*-Value
(Intercept)	1.22	0.88 to 1.56	<0.01
Heifer calving intensity (log transformed) ^‡^	−0.16	−0.30 to 0.009	0.039
IgG in the first week of life (mg/mL)	−0.01	−0.019 to 0	0.064

^‡^ Heifer calving intensity represents the number of calves born per farm per week.

**Table 3:** Results of the generalised linear mixed model for variables associated with the risk of dairy heifers (*n* = 492 on 11 farms) showing clinical signs of bovine respiratory disease (BRD) in their first nine weeks of life. Results are exponentiated odds ratios.

Variable	Odds Ratio	95% CI	*p*-Value
(Intercept)	10.79	2.25 to 51.77	0.003
Breed—Holstein	(ref)		
Breed—Other	0.63	0.33 to 1.1	0.097
Group management—continuous flow	(ref)		
Group management—fixed group	0.42	0.17 to 0.96	0.040
Mean temperature in month of birth (°C, log transformed)	0.54	0.29 to 0.97	0.042
IgG at recruitment (mg/mL)	0.98	0.95 to 1	0.030

**Table 4:** Results of the linear mixed effects model for variables associated with the risk of dairy heifers (*n* = 492 on 11 farms) showing clinical signs of bovine respiratory disease (BRD) in their first nine weeks of life.

Variable	Estimate	95% CI	*p*-Value
(Intercept)	6.02	4.17 to 7.75	<0.01
Group management—continuous flow	(ref)		
Group management—fixed group	−1.14	−2.07 to −0.21	<0.01
Mean temperature in month of birth (°C, log transformed)	−0.93	−1.56 to −0.22	<0.01
Total milk solids fed preweaning (kg)	−0.031	−0.051 to −0.012	0.002
IgG at recruitment (mg/mL)	−0.025	−0.056 to −0.014	0.001
IGF-1 at recruitment (ng/mL, log transformed)	−0.389	−0.617 to −0.176	0.001

**Table 5:** Results of the linear mixed effects model for the composite umbilical infection score for dairy heifers (*n* = 492 on 11 farms) in their first nine weeks of life.

Variable	Estimate	95% CI	*p*-Value
(Intercept)	0.044	0.03 to 0.38	0.008
Heifer calving intensity (log transformed)	0.071	0 to 0.14	0.049
Mean temperature in month of birth (°C, log transformed)	0.222	0.08 to 0.36	0.002
IGF-1 at recruitment (ng/mL, log transformed)	−0.099	−0.15 to −0.05	<0.001

## Data Availability

The data are contained within the article and in Supplementary Table S1. No additional data are available.

## Supplementary Materials

The following are available online at https://www.mdpi.com/2076-2615/11/2/378/s1, Table S1: Summary of all the variables tested for inclusion in the disease models. All normally distributed data are presented as mean ± SD. Other continuous data are presented as median and interquartile range. Categorical data are presented as count (*n*) and percentage of calves in each group.
