# Supplementary material for: A Cohort Study Risk Factor Analysis for Endemic Disease in Pre-Weaned Dairy Heifer Calves
Source: Animals (Basel). 2021 Feb 2;11(2):378. doi: 10.3390/ani11020378 (PMC7913234; doi:10.3390/ani11020378)
Supplement: Supplementary file 1 [file animals-11-00378-s001.pdf]

Supplementary Table 1. Summary of all the variables tested for inclusion in the disease models. All normally distributed data are presented as mean ± SD. Other continuous data are presented as median and interquartile range. Categorical data are presented as count (n) and percentage of calves in each group.

Variable	Descriptive statistics	Final variable tested for inclusion in models
Dam and calving related variables		
Dam perinatal disease	34 (6.9 %) of cows had perinatal disease of which: milk fever, n=22; mastitis, n=5; metritis, n= 3; retained fetal membranes, n= 3; unknown, n=1	Binary Y/N
Dystocia	68 (13.8 %) of calvings were assisted of which: manual assistance, n=42; traction, n=25; uterine torsion, n= 1	Binary Y/N
Calf breed	Holstein, n=351 (71.3%); Scandinavian Red X B&W, n=43 (8.7 %); Friesian, n=37 (7.5 %); Jersey, n=24 (4.9 %); Friesian X Jersey, n=13 (2.6%); Jersey X Other, n=11 (2.2 % %); Ayrshire, n=9 (1.8 %); Friesian X Other, n= 4 (0.8 %)	Categorical variable (all breeds) or<br>Binary (Holstein/Other)
Calving pattern	All year round pattern, 8 farms, 339 calves (68.9%); autumn block calving, 3 farms, 153 calves (31.1%)	Categorical
Calf husbandry variables		
Heifer calving intensity	The number of heifers born on that farm in the same week as the calf's recruitment was not normally distributed. The group size varied from 1 – 21, with a median of 6 and interquartile range of 3-12. Log transformation produced a normally distributed variable with a mean of 1.7 ± 0.8	Log transformation of continuous data
Mean temperature in the month of birth	This ranged from 4.4°C (February) to 16.6°C (August) and was not normally distributed. The median was 9.5°C with an interquartile range of 5.9 – 13.2°C. Log transformation produced a normally distributed variable with a mean of 2.2 ± 0.41 log°C	Log transformation of continuous data
Movement pre-weaning	288 (58.5 %) calves did not move farm pre-weaning	Binary Y/N

Age at movement	Of those that did move, age at movement was not normally distributed with a range of 1 – 62 days, a median of 17.7 days and an interquartile range of 14 – 23 days	As fewer than half of calves were moved this was not tested
Group size (as a continuous and categorical variable)	62 calves (12.6 %) in single pens; 131 (26.6 %) in small group (<7 calves); 135 (27.4 %) in medium group (7-12 calves); 164 (33.3 %) in large group (>12 calves)	Categorical Binary
Housing management	337 (68.5%) calves remained in a fixed group; 155 (31.5%) calves changed groups, termed continuous flow.	Binary (fixed group or continuous flow)
Age at disbudding	Three groups were used as categorical variables: < 3 weeks, n=14 (2.8%); 3 - 6 weeks, n=237 (48.2%); > 6 weeks, n=241 (49.0%)	Categorical
Previous disease variables		
Previous diarrhoea	255/492 (48.2%) had a clinical score indicating diarrhoea	Binary Y/N. Only included as potential risk factor for BRD.
Calf feeding variables collected on an individual calf basis		
Additional colostrum feeding	173 (35.2 %) calves supplemented	Binary Y/N
Age at separation from dam	This ranged from 0 days (snatched at birth) to 18 days and data were not normally distributed. The median time was 0.9 days with an interquartile range of 0.4-1.6 days. On several farms these data were not accurately collected as calf time of birth was uncertain.	Due to inaccuracies, the farmer’s estimate for this variable was used to calculate feeding rather than as a candidate variable
Feeding frequency	Automated feeders (all allocated 3 feeds/day), n=206 (41.9%); twice daily, n=200 (40.7 %); once daily from 3 weeks, n=86 (17.4%).	Categorical
Mixing rate	Two farms fed waste milk (n=96 calves, 19.5%). All other animals fed milk replacer at 15% concentration, n=71 (14.4 %); 12.5% concentration, n=147 (29.9%); or 10% concentration, n=274 (55.7%).	Since 20 % of calves were not fed milk replacer these values were used to calculate estimated dry matter intake rather than tested for inclusion in the model

Age at weaning	This was normally distributed with a mean age of 64.3 ± 9.4 days	Continuous<br>N.B. this was not tested for diarrhoea as the cases occurred early in life
The feeding data listed above were used to calculate the following variables on an individual calf basis		
Milk solids fed days 1-35	Normally distributed with a mean of 20.5 ± 5.0 kg	Continuous
Milk solids fed days 35 – 63	Normally distributed with a mean of 15.2 ± 5.4 kg	Continuous<br>N.B. this was not tested for diarrhoea as cases occurred early in life
Milk solids fed days 1-63	Normally distributed with a mean of 34.5 ± 10.8 kg	Continuous<br>N.B. this was not tested for diarrhoea as cases occurred early in life
Variables relating to calf morphology at recruitment		
Height at recruitment	Normally distributed with a mean of 76.4 ± cm	Continuous
Length at recruitment	Normally distributed with a mean of 61.9 ± 4.6 cm	Continuous
Weight at recruitment	Normally distributed with a mean of 39.6 ± 8.8 kg	Continuous
Ponderal index at recruitment	Normally distributed with a mean of 14.8 ± 2.4 kg/m3	Continuous
Age at recruitment	Normally distributed with a mean of 4.5 ± 2.9	Included in models because of varying age at recruitment – tested for interaction with morphological variables
Calf variables from serum and plasma testing		
Serum total protein at recruitment	Normally distributed with a mean of 56.7 ± 103 mg/ml	Continuous
Serum IgG at recruitment	Normally distributed with a mean of 19.0 ± 9.9 mg/ml	Continuous
Plasma IGF-1 at recruitment	Data were not normally distributed. The range was <1.8 – 177 ng/ml, with a median of 47 ng/ml and an interquartile range of 27.8 – 74.7	Log transformation of continuous variable, with 1 added to all values (to

	ng/ml. Log transformation produced a normally distributed variable with a mean of 3.7 ± 0.98	account for some undetectable results)
Plasma IGF-1 at week 5 (28-35 days old)	Data were not normally distributed, the range was <1.8 – 560 ng/ml with a median of 52.4 ng/ml and an interquartile range of 31.8 – 76.9 ng/ml. Log transformation produced a normally distributed variable with a mean of 3.9 ± 0.8.	Log transformation of continuous variable with 1 added to all values (to account for some undetectable results)
